# A lack of association between elevated serum levels of S100B protein and autoimmunity in autistic children

**DOI:** 10.1186/1742-2094-9-54

**Published:** 2012-03-16

**Authors:** Laila Yousef Al-Ayadhi, Gehan Ahmed Mostafa

**Affiliations:** 1 Autism Research and Treatment Center, Al-Amodi Autism Research Chair, Department of Physiology, Faculty of Medicine, King Saud University, Riyadh, Saudi Arabia; 2 Department of Pediatrics, Faculty of Medicine, Ain Shams University, 9 Ahmed El-Samman Street off Makram Ebaid, 11511 Nasr City, Cairo, Egypt

**Keywords:** Antiribosomal P protein antibodies, Autism, Autoimmunity, S100B protein

## Abstract

**Background:**

S100B is a calcium-binding protein that is produced primarily by astrocytes. Increased serum S100B protein levels reflect neurological damage. Autoimmunity may have a role in the pathogenesis of autism in some patients. Autoantibodies may cross the blood-brain barrier and combine with brain tissue antigens, forming immune complexes and resulting in neurological damage. We are the first to investigate the relationship between serum levels of S100B protein, a marker of neuronal damage, and antiribosomal P protein antibodies in autistic children.

**Methods:**

Serum S100B protein and antiribosomal P antibodies were measured in 64 autistic children in comparison to 46 matched healthy children.

**Results:**

Autistic children had significantly higher serum S100B protein levels than healthy controls (*P *< 0.001). Children with severe autism had significantly higher serum S100B protein than patients with mild to moderate autism (*P *= 0.01). Increased serum levels of antiribosomal P antibodies were found in 40.6% of autistic children. There were no significant correlations between serum levels of S100B protein and antiribosomal P antibodies (*P *= 0.29).

**Conclusions:**

S100B protein levels were elevated in autistic children and significantly correlated to autistic severity. This may indicate the presence of an underlying neuropathological condition in autistic patients. Antiribosomal P antibodies may not be a possible contributing factor to the elevated serum levels of S100B protein in some autistic children. However, further research is warranted to investigate the possible link between serum S100B protein levels and other autoantibodies, which are possible indicators of autoimmunity to central nervous system in autism.

## Introduction

S100 proteins comprise a multitude of low-molecular-weight, calcium-binding proteins that interact with other proteins to modulate biological processes [1]. They have been named "S100" because of their biochemical property of remaining soluble after precipitation with 100% ammonium sulfate [2]. S100B protein is characterized by the presence of two calcium binding sites of the EF-hand type (helix-loop-helix), one of which is located in the NH_2 _terminus and is noncanonical, whereas the other binding site is located in the COOH terminus and is canonical. This configuration enables S100 protein to respond to a calcium stimulus induced by cell signaling [3]. S100B protein is chiefly found in glial cells and Schwann cells in the central nervous system (CNS) [4]. The clinical significance of S100B protein has substantially increased throughout several areas of clinical neuroscience as it can be used as a reliable and early predictor of poor physiological and cognitive neurological outcomes [5]. Serum and cerebrospinal fluid (CSF) levels of S100B protein levels are raised in some autoimmune neuropsychiatric disorders, reflecting the presence of glial cell pathology and continuing neurological damage [6-8].

Autoimmunity may play a role in autism in a subgroup of patients [9,10], as indicated by the presence of brain-specific autoantibodies in some autistic children [11-17]. These autoantibodies may cross the blood-brain barrier (BBB) and combine with brain tissue antigens, forming immune complexes that result in damage of the neurological tissue [10]. Also, there is an increase in the frequency of autoimmune disorders within autistic families [18-23]. In spite of the fact that the origins of autoimmunity in autism are unknown, in some autistic children there is an imbalance of T helper 1 (Th1)/Th2 subsets toward Th2, which are responsible for allergic response and production of antibodies [9]. Moreover, there is a strong association between autism and the major histocompatibility complex for the null allele of C4B in the class III region. This results in low production of C4B protein, leading to repeated infections, which play an important role in the development of autoimmunity [21,24,25].

Various antibodies against neuronal tissues have been discovered in immune-mediated neurological disorders. Some of these antibodies have been found to correlate with the pathomechanism of these diseases [26]. Antiribosomal P protein antibodies are one group of potentially pathogenic autoantibodies that have a specificity for the functional center of the ribosomal P proteins. These proteins are a family of highly conserved acidic phosphoproteins located primarily on the stalk of the large (60s) ribosomal subunit [27]. They bind three ribosomal proteins, identified as P0, P1 and P2 (38, 19 and 17 kDa, respectively) by recognizing a certain epitope found in those three proteins. Several possible pathogenic mechanisms for these antibodies in some autoimmune diseases include their binding to epitopes on the cell membrane surface, intracellular penetration, inhibition of protein synthesis, production of proinflammatory cytokines and induction of cellular apoptosis [28].

In this study, we aimed to investigate the relationship between serum levels of S100B protein, a marker of neuronal damage, and antiribosomal P protein antibodies as indicators of the presence of autoimmunity in a group of autistic children.

## Methods

### Study population

This cross-sectional study was conducted on 64 children with autism. They were recruited from the Autism Research and Treatment Center, Faculty of Medicine, King Saud University, Riyadh, Saudi Arabia. Patients were fulfilling the criteria of the diagnosis of autism according to the *Diagnostic and Statistical Manual of Mental Disorders, Fourth Edition *[29]. The autistic group comprised 50 males and 14 females. Their ages ranged from 5 to 12 years (mean ± SD = 8.4 ± 2.5 years).

### Exclusions criteria

The exclusion criteria were (1) patients who had associated neurological diseases (such as cerebral palsy and tuberous sclerosis) and metabolic disorders (such as phenylketonuria); (2) patients with associated allergic, inflammatory or autoimmune disorders; and (3) patients who were receiving any medications.

The control group comprised 46 age- and sex-matched, apparently healthy children (34 males and 12 females). They were the healthy older siblings of the healthy infants who attend the Well Baby Clinic, King Khalid University Hospital, Faculty of Medicine, King Saud University, Riyadh, Saudi Arabia, for routine follow-up of their growth parameters. The control children were not related to the children with autism and demonstrated no clinical findings suggestive of immunological or neuropsychiatric disorders. Their ages ranged from 6 to 12 years (mean ± SD = 9.1 ± 2.4 years). The local ethical committee of the Faculty of Medicine, King Saud University, Riyadh, Saudi Arabia, approved this study. In addition, an informed written consent statement of participation in the study was signed by the parents or the legal guardians of the studied subjects.

### Study measurements

#### Clinical evaluation of autistic patients

The evaluation of patients was based on clinical history taken from caregivers, clinical examination and neuropsychiatric assessment. In addition, the degree of the disease severity was assessed by using the Childhood Autism Rating Scale (CARS) [30], which rates the child on a scale from 1 to 4 in each of 15 areas (relating to people; emotional response; imitation; body use; object use; listening response; fear or nervousness; verbal communication; nonverbal communication; activity level; level and consistency of intellectual response; adaptation to change; visual response; taste, smell and touch responses; and general impressions). According to this scale, children who score 30 to 36 have mild to moderate autism (*n *= 30) and those with scores ranging from 37 to 60 have a severe degree of autism (*n *= 34).

#### Serum assessment of S100B protein

Serum levels of S100B protein were evaluated using an ELISA kit [31]. To increase accuracy, all samples were analyzed twice in two independent experiments to assess the interassay variations and to ensure reproducibility of the observed results (*P *> 0.05). No significant cross-reactivity or interference was observed.

#### Measurement of serum anti-ribosomal P protein antibodies

Serum total immunoglobulin G (IgG) and IgM antiribosomal P protein antibodies were measured by ELISA using ribosomal P peptide-BSA conjugate as an antigen (Nunc-Immuno Module F8 MaxiSorp; Nunc, Roskilde, Denmark). To increase accuracy, all samples were analyzed twice in two independent experiments to assess the interassay variations and to ensure reproducibility of the observed results (*P *> 0.05). No significant cross-reactivity or interference was observed.

### Statistical analysis

The results were analyzed by using a commercially available software package (StatView; Abacus Concepts, Inc, Berkeley, CA, USA). The data are presented as means ± 2 SD in addition to medians and IQRs, which are between the 25th and 75th percentiles, for parametric and nonparametric data, respectively. Student's *t*-test and the Mann-Whitney *U *test were used for comparisons between parametric and nonparametric data, respectively. A *χ*^2 ^test was used for comparison between qualitative variables of the studied groups. Spearman's ρ correlation coefficient *r *was used to determine the relationship between different variables. For all tests, *P *< 0.05 was considered significant. Patients were considered to have elevated serum S100B protein or antiribosomal P protein antibodies if their levels were above the highest cutoff values (223.3 pg/ml and 101.3 U/ml, respectively), which were the means ± 2 SD and 95th percentiles of serum S100B protein and antiribosomal P protein levels, respectively, of healthy controls.

## Results

### Serum S100B protein levels in autistic children and their relation to the degree of the severity of autism

**Figure 1:**
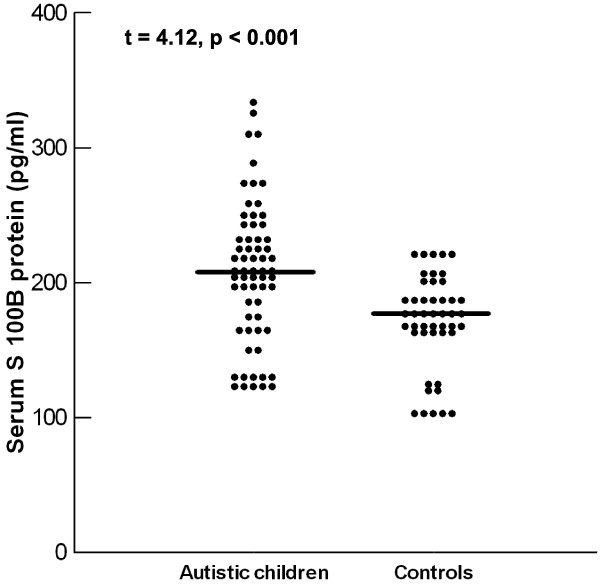
**Serum levels of S100B protein in children with autism and healthy children**. Mean values are indicated by the horizontal lines.

Autistic children had significantly higher serum S100B protein levels (207.97 ± 52.6 pg/ml) than healthy controls (171.33 ± 34.65 pg/ml) (*P *< 0.001) (Figure 1). Increased serum S100B protein levels were found in 23 (35.9%) of 64 autistic patients.

Patients with severe autism had significantly higher serum S100B protein levels (222.47 ± 52.95 pg/ml) than children with mild to moderate autism did (191.53 ± 47.89 pg/ml) (*P *= 0.01). Although the frequency of increased serum S100B protein levels was higher in children with severe autism (44.1%) than in patients with mild to moderate autism (26.7%), this difference did not reach statistical significance (*P *= 0.1) (Table 1). In spite of the presence of positive correlations between serum levels of S100B protein and CARS in autistic patients, these correlations did not reach statistical significance (*P *= 0.055).

**Table 1:** Relationship between degree of severity of autism and both elevated serum S100B protein levels and increased frequency of serum antiribosomal P protein antibodies in autistic children

Protein levels	Patients with mild to moderate autism (*n *= 30)	Patients with severe autism (*n *= 34)	P-value (χ^2 ^test)
Normal serum S100B protein (*n *= 41)	22 (73.3%)	19 (55.9%)	0.77
Elevated serum S100B protein (*n *= 23)	8 (26.7%)	15 (44.1%)	(0.27)
Normal serum antiribosomal P protein (*n *= 38)	26 (86.7%)	12 (35.3%)	17.44
Elevated serum antiribosomal P protein (*n *= 26)	4 (13.3%)	22 (64.7%)	(< 0.001)

In addition, serum S100B protein levels had no significant correlations with the age of the children with autism (*P *= 0.6).

### Relationship between elevated serum levels of S100B protein and antiribosomal P protein antibodies in autistic children

**Figure 2:**
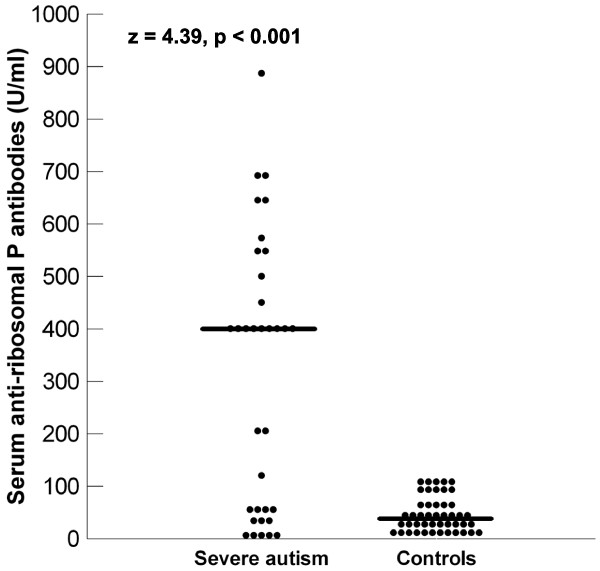
**Serum levels of antiribosomal P protein antibodies in children with severe autism and healthy children**. Median values are indicated by the horizontal lines.

**Figure 3:**
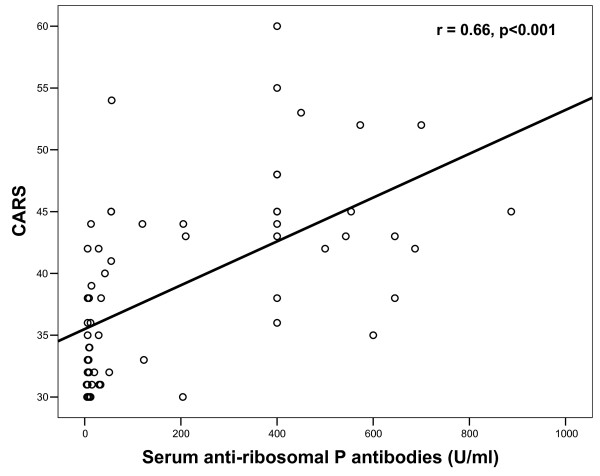
**Positive correlations between serum levels of antiribosomal P protein antibodies and CARS in children with autism**. CARS: Childhood Autism Rating Scale.

Increased serum levels of antiribosomal P protein antibodies were found in 26 (40.6%) of 64 autistic patients. Patients with severe autism had significantly higher serum antiribosomal P protein antibodies [median (IQR) = 400 (459) U/ml] than children with mild to moderate autism [median (IQR) = 9 (23) U/ml] (*P *= 0.01) (Figure 2). Also, the frequency of increased serum antiribosomal P protein antibodies was significantly higher in children with severe autism (64.7%) than in patients with mild to moderate autism (13.3%) (*P *< 0.001) (Table 1). Moreover, there were significant positive correlations between serum levels of antiribosomal P protein antibodies and CARS in autistic patients (*P *< 0.001) (Figure 3).

Although patients with elevated serum S100B protein levels had a higher frequency of positivity of serum antiribosomal P protein antibodies (47.8%) than patients with normal serum S100B protein levels did (36.6%), this difference did not reach statistical significance (*P *= 0.27) (Table 2). In addition, serum S100B protein levels had no significant correlations with serum levels of antiribosomal P protein antibodies (*P *= 0.29).

**Table 2:** Relationship between elevated serum S100B protein levels and increased frequency of serum antiribosomal P protein antibodies in autistic children

Patients with autism (*n *= 64)	Normal serum antiribosomal P protein (*n *= 38)	Elevated serum antiribosomal P protein (*n *= 26)	P-value (χ^2 ^test)
Normal serum S100B protein (*n *= 41)	26 (63.4%)	15 (36.6%)	0.77
Elevated serum S100B protein (*n *= 23)	12 (52.2%)	11 (47.8%)	(0.27)

## Discussion

S100B is an astrocytic calcium-binding protein that has been proposed as a biochemical marker of brain damage or dysfunction in acute and chronic neurological diseases [6]. In our series, autistic children had significantly higher serum S100B protein levels than healthy controls (*P *< 0.001). Increased serum S100B protein levels were found in 35.9% of autistic patients. S100B protein is a marker of neuronal damage that was first isolated from the CNS in vertebrates [2], and it is chiefly found in glial cells and Schwann cells [4]. Researchers in a previous study reported that risperidone, a drug used to improve autism symptoms, induced a statistically significant increment of about 80% of S100B protein secretion. These data contribute to the proposal that glial cells are targets of risperidone [32].

S100B is a protein produced primarily by brain astrocytes, and it is an established peripheral biomarker of altered BBB permeability associated with various CNS diseases [32,33]. Elevated S100B protein levels accurately reflect the presence of neuropathological conditions, including traumatic head injuries [33-35], psychiatric disorders [36], cerebrovascular insults [37] and neurodegenerative diseases [38], whereas normal levels reliably exclude major CNS pathology [35,39,40]. Thus the increase of serum S100B protein levels in autistic patients may indicate the presence of an underlying neuropathological condition.

The potential clinical use of measurement of serum S100B protein levels in the therapeutic decision-making process is substantiated by a vast body of literature validating variations in serum S100B levels with standard modalities for prognosticating the extent of CNS damage, alterations in neuroimaging, cerebrospinal pressure and other brain molecular markers (neuron-specific enolase, glial fibrillary acidic protein) [41,42]. The major advantage of using S100B levels is that elevations in serum can be measured easily, providing a sensitive tool with which to help rule out major CNS dysfunction. Moreover, as serum S100B levels reflect BBB permeability changes even in the absence of neuronal injury [33-39,43,44], they may increase prior to a significant change in neurological function or neuronal cell death. This is an important clinical finding, as the normal range of S100B levels rules out cerebrovascular damage and injury to the CNS in nearly 99% of patients by neurological imaging [40,45].

S100B protein is implicated in intracellular and extracellular regulatory activities. Intracellularly, it exhibits regulatory effects on cell growth, differentiation and shape, as well as energy metabolism. Extracellularly, S100B protein stimulates neuronal survival, differentiation, astrocytic proliferation, neuronal death via apoptosis and regulation of the activity of inflammatory cells [46]. S100 proteins are key mediators in polymorphonuclear neutrophil migration [47]. The degree of systemic inflammation is associated with S100B protein concentration in acute ischemic stroke [48]. Several studies have suggested that S100B protein has a role in the pathogenesis of some autoimmune neuropsychiatric diseases, such as multiple sclerosis (MS) and neuropsychiatric systemic lupus erythematosus (NPSLE) [6-8]. Phenotypically and functionally, S100B-specific T cells can be recovered from the peripheral blood of patients with MS, making S100B a potential candidate autoantigen in MS [49]. Furthermore, S100B protein may act as a cytokine [46,50,51], and *in vitro *studies have shown that, at high levels, S100B protein can induce the neuronal expression and secretion of proinflammatory IL-6. Elevated levels of S100B have been detected in the CSF of MS patients during acute phases or exacerbations of the disease [50], and it has therefore been proposed that elevated S100B protein may be indicative of active cell injury [51] and can reflect an axonal and glial pathology. Measurement of serum concentrations of S100B protein may be useful for monitoring immunosuppressive therapy and may support clinical assessment of patients with MS [8]. Serum and CSF S100B protein levels were raised in patients with NPSLE, especially among patients with organic brain syndrome, seizures, cerebral vascular accident and psychosis. This may imply that S100B protein might serve as an available and complementary biochemical marker within evaluations of NPSLE. The association of anti-double-stranded DNA (anti-dsDNA) antibodies with higher S100B protein levels may indicate that raised serum levels of S100B protein may reflect continuing neurological damage [6,7,52].

In the present work, patients with severe autism had significantly higher serum S100B protein levels than children with mild to moderate autism (*P *= 0.01). This may indicate that the extent of the elevation of serum S100B protein levels was closely linked to the degree of the severity of autism. This may be explained by the increment in the degree of neurological damage with autistic severity, resulting in more production of S100B protein. However, it is not easy to determine whether the increase in serum S100B protein levels is a mere consequence of autism or has a pathogenic role in the disease.

A possible role of abnormalities in the immune system in the pathogenesis of autism was previously postulated [9,10,53,54]. Autoimmunity to the CNS is the commonest of these abnormalities in autism. This may be indicated by the presence of brain-specific autoantibodies in some autistic children [9-17]. Immune system dysfunction may represent a novel target for treatment in autism [55]. In our series, increased serum levels of antiribosomal P protein antibodies were found in 40.6% of autistic patients. Autoantibodies are the hallmark of autoimmune diseases. We recently reported increased serum levels of antiribosomal P protein antibodies in 44.3% of another group of 70 autistic children between ages 6 and 11 years [56]. This was the only study that in which the serum levels of these antibodies in autism were investigated.

Antiribosomal P protein antibodies are highly specific for SLE, especially for the neuropsychiatric manifestations, including psychosis, mood disorders, anxiety, cognitive dysfunction and delirium [57]. A recent study has demonstrated a strong association between the seropositivity of antiribosomal P protein antibodies and the presence of neuropsychiatric manifestations in a group of children with SLE [58]. Some studies in the literature have related antiribosomal P protein antibodies to the pathogenesis of organ damage in SLE. The main pathways described are cross-reaction with anti-dsDNA antibodies, a cytotoxic effect on mesangium cell proliferation, invasion into living cells and initiation of apoptosis, a defect in the synthesis of apolipoprotein B resulting in accumulation of lipids inside the cell, and downregulation of total protein synthesis. P proteins are posttranslationally modified (dephosphorylated) during apoptosis, and a dysregulation in the normal clearance of apoptotic cells leads to aberrant exposure of the immune system to modified non-self-antigens. This could be one of the triggering events for the development of anti-P protein autoimmune response in some autoimmune diseases [57].

Moreover, in an experimental study, mice that received intracerebroventricular injections of antiribosomal P protein antibodies developed depression-like behaviors, which seems to be mediated by specific binding of these antibodies to limbic system brain areas, such as the hippocampus and the cingulate cortex. It has been proposed that antiribosomal P protein antibodies directly and/or indirectly affect the CNS and produce a cytotoxic effect on neuronal cells. The mechanism by which these antibodies cross the BBB is unknown [59].

In this work, patients with severe autism had significantly higher serum antiribosomal P protein antibodies than did children with mild to moderate autism (*P *= 0.01). Also, the frequency of increased serum antiribosomal P protein antibodies was significantly higher in children with severe autism (64.7%) than in patients with mild to moderate autism (13.3%) (*P *< 0.001). Moreover, there were significant positive correlations between serum levels of antiribosomal P protein antibodies and CARS in autistic patients (*P *< 0.001). This might indicate that the extent of the elevation of serum antiribosomal P protein antibodies was linked to the degree of severity of autism as assessed by CARS. The relationship between antiribosomal P protein antibodies and the severity of autism might be a causal one in which these autoantibodies might play a role in the pathogenesis of brain damage, the extent of which may determine the clinical severity of autism. This warrants other studies to reveal the pathogenic role of these antibodies in autism.

The reason underlying the formation of some autoantibodies in some patients with autism is not fully understood. It is speculated that an autoimmune reaction might be trigged by cross-reacting antigens in the environment, resulting in the release of some self-antigens. These antigens may result in the induction of autoimmune reactions through the activation of inflammatory cells in genetically susceptible individuals [9,10]. The involvement of the cellular redox state in the pathogenesis of autoimmune diseases, including autism, has been extensively demonstrated. Protein peroxidation confers alterations in protein structure with impairment of their functional properties. Proteins with a substantial conformational loss are potential sources of neoantigens, leading to induction of autoimmunity in a variety of autoimmune diseases [60,61]. Researchers in a recent study reported that many autistic children have increased oxidative stress resulting from enhanced lipid peroxidation and/or a decrease in the level of glutathione peroxidase, which is an important antioxidant. The same researchers in that study also reported a possible role of oxidative stress in the induction of autoimmunity in some autistic patients [62]. Moreover, one study found that CD4^+^CD25^high ^regulatory T cells were deficient in 73.3% of a group of 30 autistic children [23]. These cells play an active part in the establishment and maintenance of immunological self-tolerance and thereby prevent autoimmunity [63,64]. Thus a deficiency of these cells may contribute to autoimmunity in a subgroup of autistic children [23].

Serum S100B protein levels have been reported to be increased in some autoimmune neuropsychiatric diseases, indicating the presence of underlying neurological damage [6-8]. Because autoantibodies can cross the BBB and combine with brain tissue antigens to form immune complexes that result in neurological damage [10], we have tried to find a possible link between the elevated serum levels of S100B protein and antiribosomal P protein antibodies in autism. In this work, although patients with elevated serum S100B protein levels had a higher frequency of positivity of serum antiribosomal P protein antibodies (47.8%) than patients with normal serum S100B protein levels did (36.6%), this difference did not reach statistical significance (*P *= 0.27).

In addition, serum S100B protein levels had no significant correlations with serum levels of antiribosomal P protein antibodies (*P *= 0.29). We could not trace data in the literature to compare our results concerning the relationship between serum levels of S100B protein and autoantibodies in autistic patients. This study is the first to explore such a relationship.

The results of this study may indicate that the presence of antiribosomal P protein antibodies in some autistic children may not be a possible contributing factor to their elevated serum levels of S100B protein. The increased serum levels of S100B protein may be attributable to other factors that may participate in neurological damage in autism. However, this is an initial report that warrants further research to determine the possible link between the elevated serum levels of S100B protein and antiribosomal P protein antibodies in autistic children.

## Conclusions

S100B protein levels were elevated in autistic children, and they were significantly correlated with the degree of the severity of autism. This may indicate the presence of an underlying neuropathological condition in autistic patients. Antiribosomal P protein antibodies might not be a contributing factor to the elevated serum levels of S100B protein in some autistic children. However, further research is warranted to investigate the possible link between serum S100B protein levels and other autoantibodies, which are possible indicators of autoimmunity to CNS, in autism.

## Abbreviations

BSA: bovine serum albumin; CARS: Childhood Autism Rating Scale; CNS: central nervous system; ELISA: enzyme-linked immunosorbent assay; IL: interleukin; kDa: kilodalton; Th: T helper; SLE: systemic lupus erythematosus.

## Competing interests

The authors declare that they have no competing interests.

## Authors' contributions

Both authors designed, performed and wrote the research. In addition, both authors read and approved the final manuscript.
